# In utero human intestine contains maternally derived bacterial metabolites

**DOI:** 10.1186/s40168-025-02110-0

**Published:** 2025-05-06

**Authors:** Wenjia Wang, Weihong Gu, Ron Schweitzer, Omry Koren, Soliman Khatib, George Tseng, Liza Konnikova

**Affiliations:** 1 Department of Biostatistics, University of Pittsburgh, Pittsburgh, PA USA; 2 Department of Pediatrics, Yale School of Medicine, New Haven, CT 06519 USA; 3 Azrieli Faculty of Medicine, Bar-Ilan University, Safed, Israel; 4 Department of Biotechnology, Tel-Hai College, Upper Galilee, Kiryat Shmona, Israel; 5 Department of Natural Compounds and Analytical Chemistry, MIGAL Galilee Research Institute, Kiryat Shmona, Israel; 6 Department of Immunobiology, Yale School of Medicine, New Haven, CT USA; 7 Department of Obstetrics, Gynecology, and Reproductive Sciences, Yale School of Medicine, New Haven, CT USA; 8 Program in Translational Biomedicine, Yale School of Medicine, New Haven, CT USA; 9 Human Translational Immunology Program, Yale School of Medicine, New Haven, CT USA; 10 Center for Systems and Engineering Immunology, Yale School of Medicine, 375 Congress Avenue, New Haven, CT 06519 USA

## Abstract

**Background:**

Understanding when host-microbiome interactions are first established is crucial for comprehending normal development and identifying disease prevention strategies. Furthermore, bacterially derived metabolites play critical roles in shaping the intestinal immune system. Recent studies have demonstrated that memory T cells infiltrate human intestinal tissue early in the second trimester, suggesting that microbial components such as peptides that can prime adaptive immunity and metabolites that can influence the development and function of the immune system are also present in utero. Our previous study reported a unique fetal intestinal metabolomic profile with an abundance of several bacterially derived metabolites and aryl hydrocarbon receptor (AHR) ligands implicated in mucosal immune regulation.

**Results:**

In the current study, we demonstrate that a number of microbiome-associated metabolites present in the fetal intestines are also present in the placental tissue, and their abundance is different across the fetal intestine, fetal meconium, fetal placental villi, and the maternal decidua. The fetal gastrointestinal samples and maternal decidua samples show substantially higher positive correlation on the abundance of these microbial metabolites than the correlation between the fetal gastrointestinal samples and meconium samples. The expression of genes associated with the transport and signaling of some microbial metabolites is also detectable in utero.

**Conclusions:**

We suggest that the microbiome-associated metabolites are maternally derived and vertically transmitted to the fetus. Notably, these bacterially derived metabolites, particularly short-chain fatty acids and secondary bile acids, are likely biologically active and functional in regulating the fetal immune system and preparing the gastrointestinal tract for postnatal microbial encounters, as the transcripts for their various receptors and carrier proteins are present in second trimester intestinal tissue through single-cell transcriptomic data.

Video Abstract

**Supplementary Information:**

The online version contains supplementary material available at 10.1186/s40168-025-02110-0.

## Introduction

Establishing and maintaining a “healthy” intestinal microbiome are critical to the overall health of an individual. This is partially facilitated by bacterially derived metabolites that are important mediators of intestinal health and regulators of intestinal mucosal immunity. Furthermore, disruption of homeostasis can lead to a myriad of diseases [1, 2]. Several groups of bacterial metabolites have been identified that are particularly important to the gut homeostasis including short-chain fatty acids (SCFA), and secondary bile acids, among others [3]. Understanding how and when the dialogue between the intestinal tract (host) and the bacterial metabolites is established is important to designing new strategies in preventing disease and improving health. There has recently been a lot of attention on the development of the microbiome over the first 3 years of human life (or the first 1000 days) as being a critical window to modulate the development and adaptation of the immune system and overall health [4]. With the detection of the memory T cells within the human and nonhuman primate fetal intestines [5–9], cord blood [10], and placenta [11], among other tissues, scientists are appreciating that education of the intestinal adaptive immune system may be ongoing in utero and the critical window of shaping the intestinal homeostasis potentially starts prior to delivery [12]. Yet there are only limited studies about the antigens present in utero or the metabolites modulating the intestinal homeostasis [13, 14].

Several studies have reported that the placenta lacks a microbiome [15–19] reviewed in [20], and others report minimal bacterial colonization in fetal meconium [21, 22], as well as placental and endometrial samples [23–28]. Furthermore, research conducted by Lauder et al. [16], involving a large cohort, found no discernible difference between placental samples and kit samples (contamination introduced during DNA purification). However, pioneering work from the MacPherson’s and other groups demonstrated that metabolites from the maternal intestinal microbiome can be detected in the murine fetal intestine and alter the development of the fetal mucosal immune system [29] as well as placental, intestinal, and fetal brain development [13, 14]. Building on this, our previous study found a unique fetal intestinal metabolomic profile with an abundance of bacterially derived metabolites and aryl hydrocarbon receptor (AHR) ligands implicated in mucosal immune regulation [30].

In the current study, we hypothesized that microbiome-associated metabolites detected in fetal intestines in utero are primarily derived from the maternal microbiota and play a role in preparing the intestinal immune system for ex-utero life. To study this, we assembled a cohort of human pregnancy-matched fetal organs including the fetal intestine, the fetal meconium, and the fetal placental villi, to the maternal decidua. We found that some of the microbial byproducts or metabolites present in fetal intestines in utero were maternally derived and vertically transmitted to the fetus (abundance changes from maternal tissues to fetal intestines), while some were found to be in a steady state (where the rate of production is balanced by its rate of consumption, resulting in a stable and unchanging level).

Importantly, these bacterially derived metabolites are likely biologically active and functional in regulating the fetal immune system and prepare the gastrointestinal tract for postnatal microbial encounters as we were able to detect their receptors and carrier proteins in single-cell transcriptomic data of the human fetal small intestine.

## Results

To investigate the source of in utero intestinal metabolome, we performed untargeted metabolomic analysis, including human and bacterially derived metabolites (manually curated to be fully bacterially derived or require partial conversion by the bacteria), using in-house pipeline established by the Khatib lab [30] on 49 tissue samples from 24 subjects (with gestational age ranging from 14 to 23 weeks): 8 maternal decidua samples, 11 fetal placental villi (PV) samples, 11 fetal gastrointestinal (GI) (fetal small intestine (SI) and large intestine (LI)) samples, and 19 fetal meconium samples (Supplementary Table S1).

### Metabolite profiles within different tissues

**Fig. 1:**
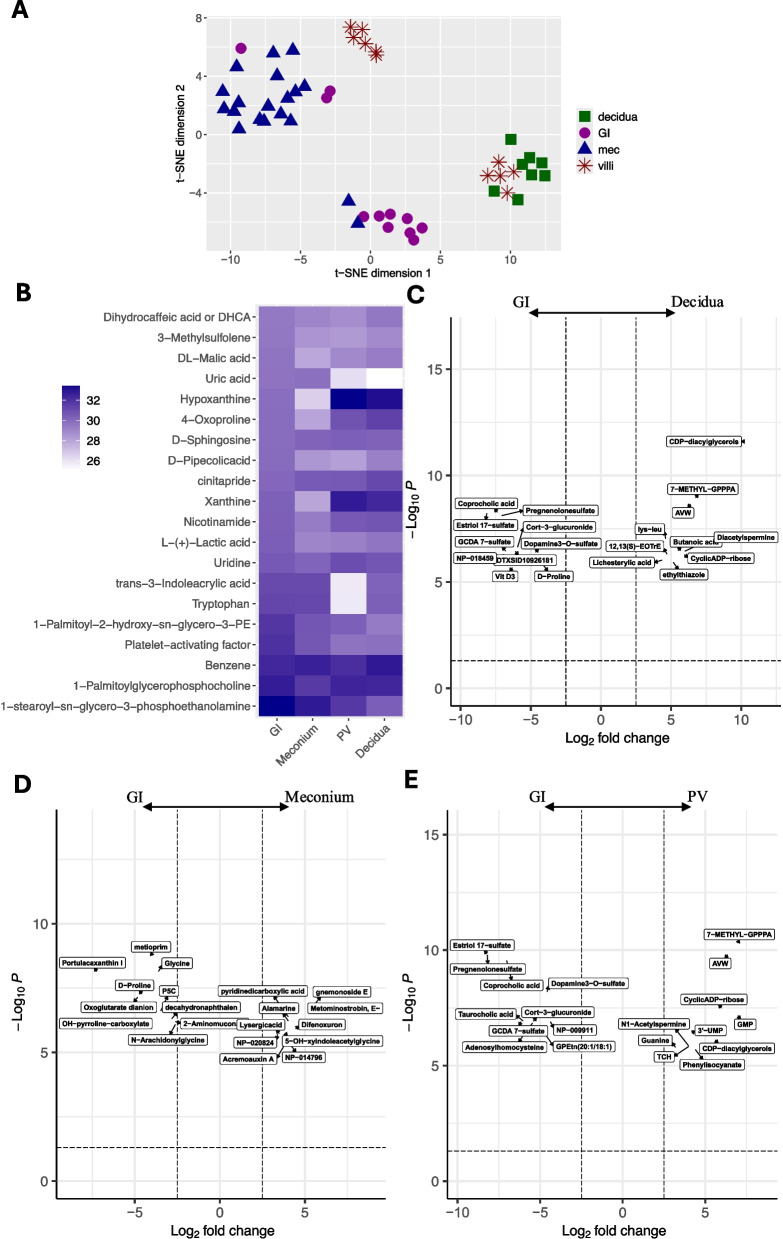
Sample separation and differential expression of individual metabolites. **A** t-distributed stochastic neighbor embedding (t-SNE) plot using all metabolites. **B** Heatmap showing normalized abundance of the top 20 abundant metabolites in the fetal GI samples (SI and LI), as well as their abundance in the meconium (SI Mec and LI Mec), PV, and maternal decidua samples. **C**, **D**, and **E** Volcano plots of differentially abundant metabolites between the GI and decidua groups, GI and meconium groups, and GI and PV groups, respectively. Top 10 significantly differentially abundant metabolites are labeled with the metabolite name

A total of 2521 metabolites were annotated in all of the 49 samples which were divided into 3 levels of annotation (9 in Level 1, 402 in Level 2, and 2110 in Level 3, see details in Supplementary Tables S2, S8, and S9). t-SNE visualization based on the 2521 analyzed metabolites showed that samples of GI, meconium (luminal contents of the small intestine), decidua, and PV were well separated, although some PV samples were mixed with decidua samples possibly due to incomplete tissue separation. To further analyze this, we also examine the separation between samples from different subjects (Supplementary Fig. S1) where we found that most of the PV samples mixed with decidua samples were in fact from the same subject. The separation indicated that even though most metabolites were detected across all samples, they were differentially abundant based on tissue source (Fig. 1A). Since our primary interest was the development of the fetal intestine and the source of bacterial metabolites in GI tract (i.e., where the bacterial metabolites present in GI tract come from), we first focused on the difference between GI tract and the other tissue groups. The top 20 GI-enriched metabolites that had differential abundances between GI and the other tissue groups are shown in Fig. 1B. To explore tissue-specific individual metabolite signatures in the decidua, PV, and meconium groups, we performed differential metabolite abundance analysis between GI and each of the other groups (see details in Supplementary Table S3). We detected a large proportion of metabolites whose abundance differed between tissue: GI vs. decidua = 326, 13% (Fig. 1C); GI vs. meconium = 318, 13% (Fig. 1D); and GI vs. PV = 324, 13% (Fig. 1E). Specifically, among the 326 differentially abundant metabolites between GI and decidua tissue, more than half (195 metabolites) were enriched for in the GI tissue, 2 of which were bacterial metabolites (coprocholic acid and glycodeoxycholic acid, secondary bile acids). For the 131 metabolites significantly enriched in decidua, 5 were bacterial metabolites (pyridoxine, methylhippuric acid, hippurate, 2-hydroxyhippuric acid, and indoxyl sulfate). In the comparison between the GI and meconium samples, 164 metabolites had higher abundance in GI tissue including 3 bacterial metabolites (N-acetyl-alpha-D-glucosamine1-phosphate, kynurenine, and phosphopantothenic acid). Interestingly, two bacterial metabolites (benzoate and albaflavenol) were enriched for in meconium. At the same time, there were six xenobiotics (nifedipine, perindopril, 2-{2-[2-(decyloxy)ethoxy]ethoxy}ethanol, picaridin, 18-acetoxy- 1alpha-hydroxyvitamin D3, ketorolac) enriched for in the meconium, suggesting that even maternally ingested compounds can be concentrated in the meconium. Comparisons between the GI and PV tissue identified 235 metabolites that were enriched for in the GI tissue, with 2 bacterial metabolites (coprocholic acid and glycodeoxycholic acid), 4 primary bile acids (glycocholic acid, 7-sulfocholic acid, taurocholic acid and taurochenodeoxycholic acid), 1 aromatic amino acid (D-(+)-tryptophan), and 2 xenobiotics (adaprolol and elacytarabine). Among the 89 metabolites with a higher abundance in the PV tissue, there were 2 bacterial metabolites (methylhippuric acid and indoxyl sulfate) and 2 xenobiotics (penicillin-G and a THC derivative).

### Metabolic pathways enriched in different tissues

**Fig. 2:**
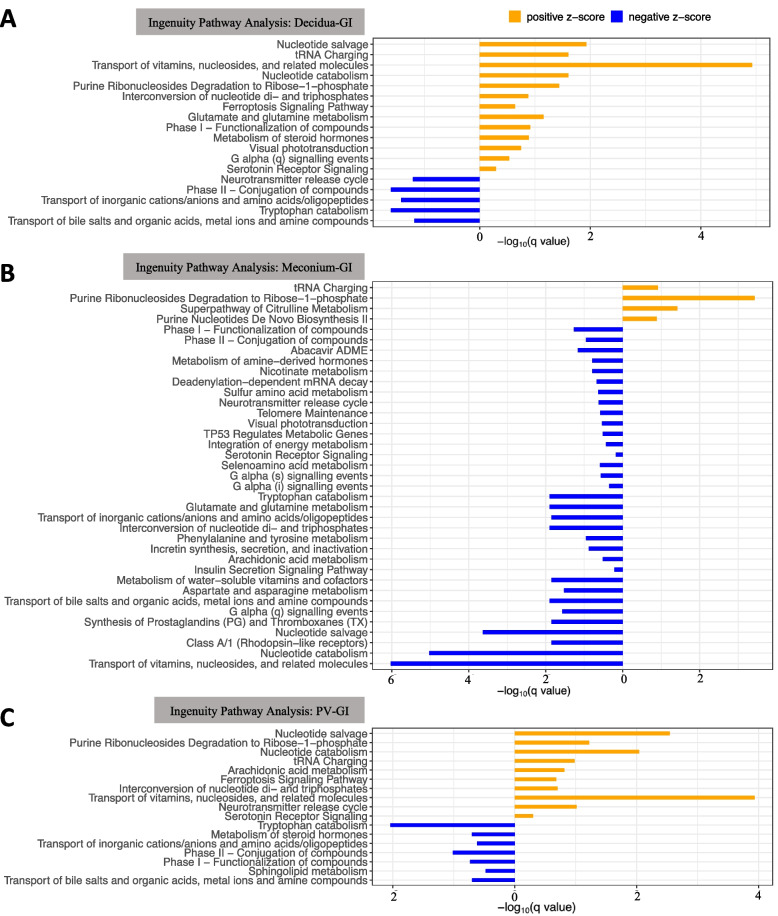
Pathway enrichment. **A**, **B**, and **C** Integrated pathway analysis for differentially altered pathways between GI and decidua groups, between GI and meconium groups, and between GI and PV groups. The length of the bar is proportional to the *q*-value. Pathways with positive values on the *x*-axis (orange bar) are those enriched for in the decidua, meconium, and PV, respectively. Those with negative values (blue bars) are pathways enriched for in the GI samples

To understand which pathways are differentially regulated in different tissues, we conducted Ingenuity Pathway Analysis (IPA) (see details in Supplementary Table S4). Of the pathways that were significantly altered between the decidua and GI samples, majority were more activated in the decidua, including the following: the transport of vitamins and steroid metabolism consistent with known functions of the placenta, while tryptophan catabolism and bile acid transport were more active in the GI track (Fig. 2A). Very few pathways were more active in the meconium, as expected as it is primarily composed of apoptotic cells. Interestingly, pathways classically associated with postnatal intestinal epithelial function were already enriched for in the GI samples prenatally including upregulation of many metabolism and transport pathways, prostaglandin synthesis, and neurotransmitter release (Fig. 2B). Our group had previously discovered that human fetal intestinal cells can produce insulin and respond to glucose concentrations [31]; in support of this, the current data identified that insulin secretion was upregulated in the fetal GI tract (Fig. 2B). Bile acid transport pathways were also upregulated in the GI samples compared to PV samples (Fig. 2C).

### Source of microbial metabolites in the GI tract

**Fig. 3:**
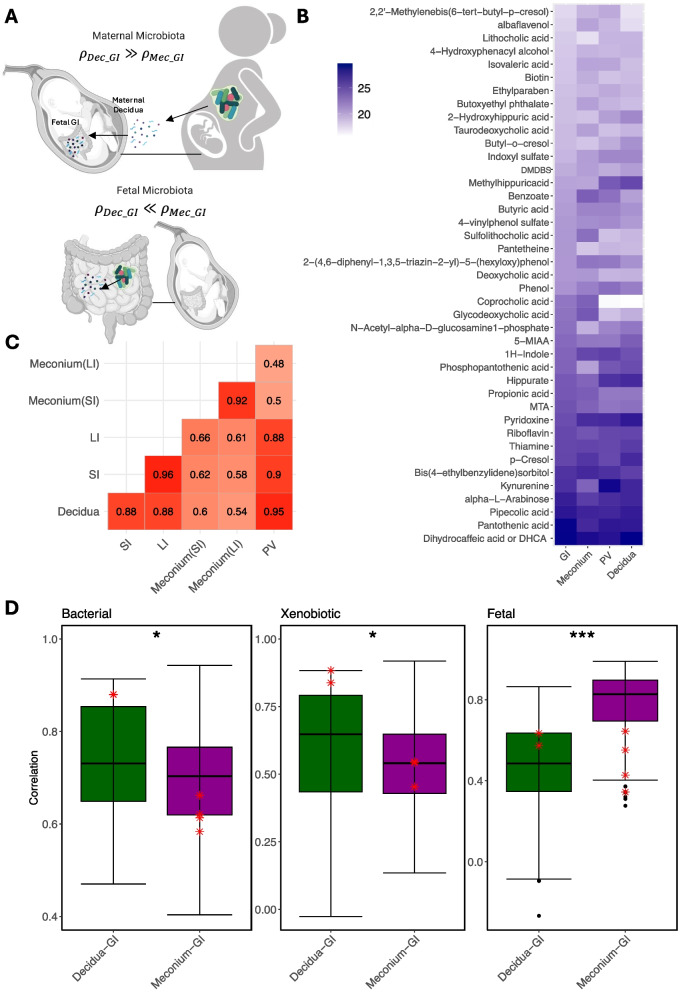
Correlation between tissue groups of microbial metabolites, xenobiotics, and fetal metabolites. **A** Schematic of how the correlation between tissue would identify the source of bacterial metabolites. ${\rho }_{Dec\_GI}$ denotes the correlation of metabolite abundance in between decidua and GI tissues, and ${\rho }_{Mec\_GI}$ denotes the correlation of metabolite abundance between meconium and GI tissues. Maternal microbiota-derived metabolites that cross as metabolites are expected to have ${\rho }_{Dec\_GI}$ $\gg {\rho }_{Mec\_GI}$, while metabolites that are potentially locally produced by fetal microbiota within the GI track are expected to have ${\rho }_{Dec\_GI}$ $\ll$ ${\rho }_{Mec\_GI}$. **B** Heatmap showing normalized abundance of the 41 microbial-associated metabolites across sample types. **C** Pairwise correlation matrix of the 41 microbial metabolites between paired tissue samples from subject no. 16. **D** Boxplots visualizing the correlations between GI and decidua groups and between GI and meconium groups based on 41 microbial metabolites, 47 xenobiotics, and 8 fetal-derived metabolites respectively from Supplementary Table S4. Red asterisk points represent the pairwise correlations between tissue samples from subject no. 16. *P < 0.05, ***P < 0.001

To understand the source of the bacterial metabolites present within the fetal intestine and determine if they are vertically transmitted from the maternal microbiota, we performed correlation analysis between the fetal intestinal tissue and the decidual tissue (maternal origin) and fetal intestine and the meconium (local production, Fig. 3A). Among the 2521 metabolites, we identified and selected 41 microbially derived or bacteria-associated metabolites of interest based on a literature search [30, 32, 33], including 5 secondary bile acids, 3 short-chain fatty acids (SCFA), and 3 aromatic lactic acids (Supplementary Table S9). In addition, we identified 47 xenobiotics, metabolites that cannot be produced in the human host, and 8 metabolites enriched for in the fetal tissue (Supplementary Tables S5, S9). All bacterial metabolites were present in the GI tissue, but the four tissues had different signatures of these metabolites (Fig. 3B). To understand the source of the metabolites better, we used the matched tissue from one subject (i.e., the tissue samples of all sites collected from one subject) to perform correlation analysis. As expected, the SI and LI and the decidua and PV samples that come from adjacent tissue had the highest correlation values of all samples, validating our analysis. Interestingly, the paired fetal GI and decidua samples had substantially higher positive correlation than the paired GI and meconium samples in terms of the abundance of the 41 microbial metabolites: ${\rho }_{Dec\_SI}=0.88 and {\rho }_{Dec\_LI}=0.88$ while ${\rho }_{MecSI\_SI}=0.62, { \rho }_{MecLI\_SI}=0.58, and {\rho }_{MecSI\_LI}=0.66, {\rho }_{MecLI\_LI}=0.61$ (Fig. 3C), suggesting that the microbial metabolites detected in fetal samples were more likely to be vertically transmitted from maternal microbiota. To determine if this positive correlation also held true for all the samples in the study, we performed the two correlative analysis (GI/decidua and GI/meconium) using all samples combined (88 pairs of GI/decidua samples and 209 pairs of GI/meconium samples across all subjects) for xenobiotics (expecting the ratio of GI/decidua to be more positively correlated than GI/meconium as these can only come from maternal circulation), fetal-derived metabolites (expecting the ratio of GI/decidua to be less than GI/meconium as these are locally produced in the fetus), and of microbiome-associated metabolites. As expected, there was a significantly higher correlation based on the 47 xenobiotics between GI samples and decidua samples compared to the corresponding correlation between GI samples and meconium samples (Fig. 3D). There was also a lower correlation based on the eight fetal-derived metabolites between GI samples and decidua samples compared to the corresponding correlation between GI samples and meconium samples (Fig. 3D). Consistent with data from Fig. 3C from an individual subject, we also observed that bacterially derived metabolites had a higher correlation between GI samples and decidua samples compared to the corresponding correlation between GI samples and meconium samples (Fig. 3D). Since the tissue samples were from subjects of different gestational ages, the correlation analysis using all samples combined might have been affected by the gestational age (though the gestational ages were balanced across samples of different tissues as demonstrated in Supplementary Fig. S2, without any statistically significant differences). Thus, we constrained each pair of samples to come from the subjects with the same gestational age, 23 weeks, and compared the correlations of GI/decidua (12 pairs) and GI/meconium (21 pairs) with the similar boxplots (Supplementary Fig. S3). Again, the correlation of bacterial metabolites and the correlation of xenobiotics were overall higher for GI/decidua than GI/meconium pairs, while the correlation of fetal-derived metabolites had the opposite trend. However, limited by the small number of sample pairs, the correlation differences were no longer statistically significant.

**Fig. 4:**
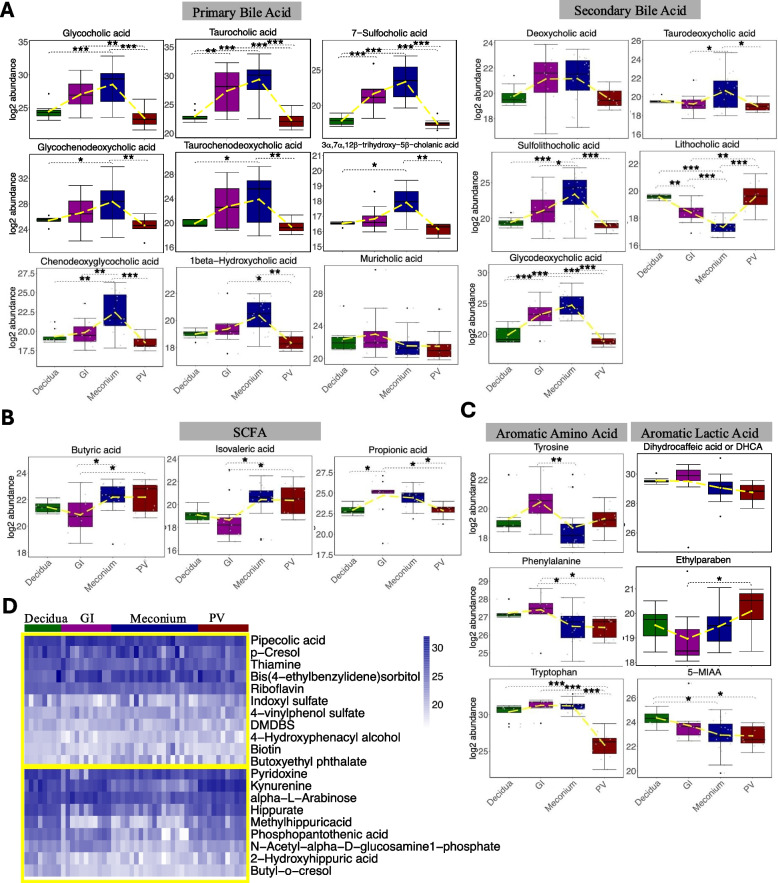
ANOVA analysis of SCFA, bile acids, and aromatic acids across tissues. **A**, **B**, **C** Boxplots of individual metabolite’s abundance for primary and secondary bile acids, SCFA, and aromatic amino acids and aromatic acids. *P < 0.05, **P < 0.01, ***P < 0.001. **D** Heatmap showing that microbial metabolites without significant difference across tissue groups (upper panel) and microbial metabolites significantly enriched in decidua samples (lower panel)

Overall, the abundance of the 41 microbial metabolites identified in our dataset demonstrated a high correlation between the GI and decidua samples. To determine if there was variability by individual metabolite, we determined the abundance enrichment across the tissues for each metabolite. In our analysis, the primary bile acids that are produced in the fetal liver and then absorbed by the small intestine, as expected, were significantly enriched for in fetal meconium but deprived in maternal decidua except for muricholic acid whose abundance was similar across tissues (Fig. 4A). Upon crossing the intestinal lumen, secondary bile acids are produced by microbiota-mediated dehydroxylation or deconjugation of the bile acids [34]. We were able to identify five secondary bile acids present in our dataset. Lithocholic acid was enhanced for in the decidua, perhaps indicating that it was produced by maternal microbiota, while deoxycholic acid was present in similar abundance across all the tissues. Surprisingly, our analysis identified three secondary bile acids: glycodeoxycholic acid, sulfolithocholic acid, and taurodeoxycholic acid whose abundances were high in meconium samples. Considering that the samples were from different subjects with different gestational ages, we compared the distributions of the gestational age within the four tissue groups (Supplementary Fig. S2) and further examined whether the abundance difference of each bile acid across the tissues was related to the fetal age (Supplementary Table S6). The results of both analyses confirmed that the gestational ages are balanced across samples of different tissues, and the fetal age does not have a significant impact on the abundance difference for any bile acid.

We then determined if short-chain fatty acids (SCFA), an important source of nutrition for intestinal epithelial cells and modulators of immunity, which are usually locally produced within the intestinal lumen, were present in the fetal intestine. Three SCFA were detected in our dataset, butyric acid was annotated as level 1, while the other two were annotated as Level 3 based on MS1 (Supplementary Tables S8, S9) and will require more validation in the future. Among them, butyric acid and isovaleric acid were significantly reduced in the GI tissues and enriched in the meconium and the PV samples (Fig. 4B). Butyric acid is an important energy source for enterocytes [35, 36], which probably explains why its level was reduced in the GI tissues compared to the others, while propionic acid was enriched in the GI samples above all other tissues (Fig. 4B).

Another important group of bacterially derived metabolites is aromatic lactic acids. Breastfeeding has been reported to promote *Bifidobacterium* species converting aromatic amino acids tyrosine, phenylalanine, and tryptophan into their respective aromatic lactic acids dihydrocaffeic acid (DHCA) and ethylparaben and 5-MIAA (5-methoxyindoleacetate) that are biologically active in the intestine and are associated with anti-inflammatory properties [32, 33]. We additionally explored if these metabolites are present in fetal tissue. The three aromatic amino acids were similarly abundant in the decidua and the GI tract with tryptophan levels being reduced in the meconium (Fig. 4C). All three of the aromatic lactic acids were present in fetal tissue, where 5-MIAA level was lowest in the meconium, and the levels of DHCA and ethylparaben were similarly abundant between the decidua, GI, and meconium samples (Fig. 4C).

Finally, we evaluated the remaining bacterially associated metabolites present in fetal tissue. Nine of these microbial metabolites (pyridoxine, N-acetyl-alpha-D-glucosamine1-phosphate, alpha-L-arabinose, kynurenine, methylhippuricacid, phosphopantothenic acid, hippurate, butyl-o-cresol, and 2-hydroxyhippuric acid) were significantly enriched for in decidua samples, while 11 microbial metabolites (riboflavin, pipecolic acid, 4-hydroxyphenacyl alcohol, 4-vinylphenol sulfate, 1,3:24-bis (3,4-dimethylobenzylideno) sorbitol (DMDBS), biotin, butoxyethyl phthalate, bis(4-ethylbenzylidene)sorbitol, thiamine, p-Cresol, and indoxyl sulfate) were found to be similarly abundant across all tissue samples (Fig. 4D).

To ensure that metabolites were identified correctly, a number of them were validated with known standards. These included butyric acid, deoxycholic acid, pantetheine, p-Cresol, taurochenodeoxycholic acid, hippurate, benzoate, and taurocholic acid (Supplementary Fig. S4). In some case where samples and where no additional samples were available, metabolites were validated by standards, but the exact concentrations of the samples could not be calculated (Supplementary Fig. S4). Where additional samples were available, metabolites were validated by standards, and exact concentrations were calculated (Supplementary Fig. S4). For deoxycholic acid, both methods were used. The results demonstrated consistent trends between the targeted and untargeted analysis among the four tissue groups for each metabolite except hippurate where quantitative analysis did not identify any difference in its abundance between groups.

### Metabolite associations with gestational ages

Intestinal bile acids have been shown to alter abundance and type of mucosal regulatory T cells. Interestingly, T cells begin to populate the small intestine in humans early on in the second trimester [37], suggesting that the differential presence of various bile acids across gestational ages may be important in establishing/regulating mucosal immunity. Similarly, SCFA have also been shown to play a role in mucosal immunity, particularly T-cell homeostasis [38]. Here, we explored the association between the abundance of the nine identified primary bile acids, five secondary bile acids, and three SCFAs with gestational age within tissue groups. Three primary bile acids: muricholic acid, 1beta-hydroxycholic acid, and 3a,7a,12b-trihydroxy- 5b-cholanic acid had a significant positive association with advancing gestational age in the GI tissue, indicative of their increased synthesis in the fetus with advancing gestational age (Table 1). In contrast, although most of the bile acids and SCFAs did not have any significant association with the gestational age of the samples (Supplementary Table S7), we found that two secondary bile acids, deoxycholic acid and glycodeoxycholic acid, and one SCFA, propionic acid, had a significant negative correlation with advancing gestational age in the fetal intestine (Table 1). The negative correlation suggests these compounds decrease in the GI tract with increasing gestational age, highlighting that they are likely coming from maternal circulation rather than local production, since if the compounds were produced locally, they would be more abundant in GI tract as the pregnancy progressed.

**Table 1:** Correlation coefficients between the abundance of individual metabolites and the gestational age within each tissue group. Here we only show the five bile acids and one SCFA (*p*ropionic acid) with significant age association. The rows are the correlation coefficient within each tissue group. The q*-*value for the correlation coefficient is show*n* in par*en*thesis

	**Deoxycholic acid**	**Glycodeoxycholic acid**	**Propionic acid**	**Muricholic acid**	**1beta-Hydroxycholic acid**		**3α,7α,12β-trihydroxy- 5β-cholanic acid**
Decidua	− 0.33 (0.94)	− 0.38 (0.94)	− 0.15 (0.94)	0.37 (0.94)	0.26 (0.94)	0.09 (0.94)	
GI	− 0.29*** (< 0.001)	− 0.19* (0.05)	− 0.25*** (< 0.001)	0.45*** (< 0.001)	0.35*** (< 0.001)	0.26*** (< 0.001)	
Meconium	0.28 (0.43)	0.29 (0.43)	0.07 (0.76)	− 0.24 (0.43)	0.09 (0.73)	0.07 (0.73)	
PV	− 0.29 (0.96)	− 0.16 (0.96)	0.24 (0.96)	0.21 (0.96)	0.10 (0.96)	0.03 (0.96)	

^*^ *P* < 0.05, **P < 0.01, ***P < 0.001

### Biological function of microbial metabolites in utero

**Fig. 5:**
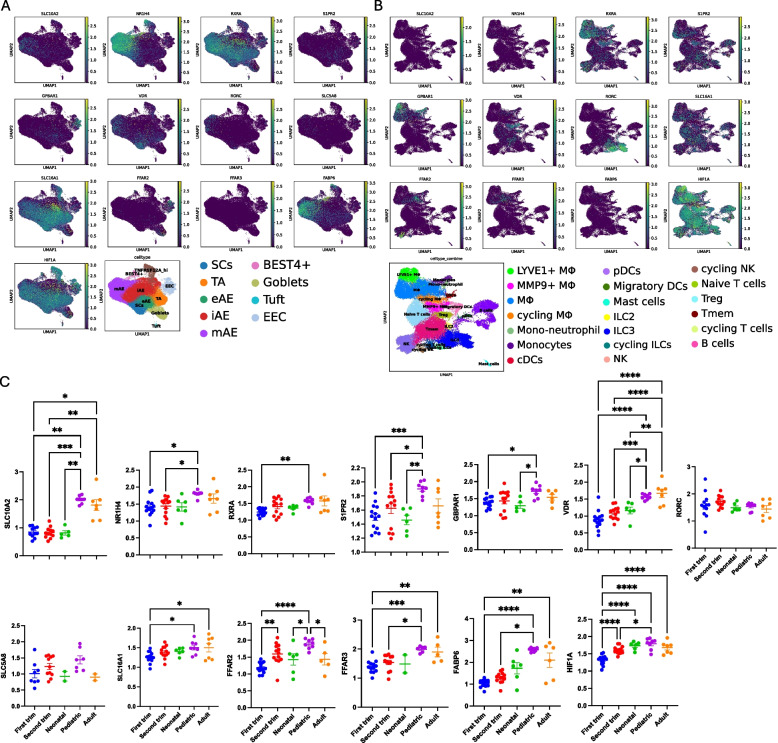
Cell type-specific expression of genes associated with bile acid and SCFA transport and signaling. scRNAseq data from our previous published manuscript [39] (Fig. 1, Supplementary Table 1; first trimester (8- to 13-week gestational age) *n* = 14, second trimester (14- to 23-week gestational age) *n* = 13, neonatal (1–14 days old) *n* = 6, pediatric (4–12 years old) *n* = 8, and adult (25–70 years old) *n* = 7). Uniform manifold approximation and projection (UMAP) plot visualization of bile acid and SCFA-associated transport and signaling gene expression in fetal small intestine epithelial (**A**) and fetal immune cells (**B**). **C** Boxplots of the mean gene expression value of positive cells in each sample from the small intestine across developmental stages (related to Fig. 1 [33]). Each dot represents an individual sample. Data are presented as mean ± SEM. *P < 0.05, **P < 0.01, ***P < 0.001. Clarification of cell type abbreviation for epithelial lineage cells in **A**: SCs, stem cell; TA, transit-amplifying cells; eAE, early enterocytes; iAE, intermediate enterocytes; mAE, mature enterocytes; EEC, enteroendocrine cells. Immune lineage cells in **B**: Mϕ, macrophages; cDCs, conventional dendritic cells; ILC, innate lymphoid cells; NK, natural killer cells; Treg, regulatory T cells; Tmem, memory T cells

We have recently developed a single-cell atlas combining a number of datasets to build a comprehensive atlas of the small intestine across the human life span including during gestation [39]. To determine if bacterially derived metabolites such as SCFA and secondary bile acids that we found in the fetal small intestine can have a biological function in utero, we explored this atlas for expression of genes associated with bile acids transport and signaling ( *SLC10 A2,* a gene that encodes apical sodium-dependent bile acid transporter (ASBT), the gene *NR1H4, a gene* that encodes farnesoid X receptor (FXR), *RXRA, a gene* that encodes retinoid X receptor alpha (NR2B1), as well as *S1PR2*, *GPBAR1*, *VDR*, and *RORC* genes) and SCFA transport and signaling (*SLC5A8* (SCFA transporter), *SLC16A1*, a gene that encodes monocarboxylate transporter 1 (MCT1), *FFAR2*, *FFAR3*, and *FABP6, a gene* that encodes intestinal bile acid binding protein (iBABP), and *HIF1A*) (Fig. 5 and Supplementary Fig. S5). The expression of *SLC10 A2*, an apical sodium-dependent bile acid transport carrier, significantly increased post-delivery, with minimal expression in utero. However, the expression of several bile acid and SCFA-associated genes was present in utero and restricted to subtypes of epithelial (Fig. 5A and Supplementary Fig. S5 A) and or immune cells (Fig. 5B and Supplementary Fig. S5B) within the SI. The expression of *VDR* and *FABP6* steadily increased from the first trimester through adulthood with highest levels present in adults (Fig. 5C). Nevertheless, it was still detectable in utero, particularly in the second trimester. The *FABP6* was expressed exclusively in the intestinal epithelial cells (IEC) within the mature absorptive (mAE) subtype, and *VDR* was expressed both in the IEC (stem cells (SCs) and mAE cells) and in the immune cells (cycling ${\text M}\upphi$, Tregs, and memory CD4 cells). Several other genes (*GPBAR1*, *SLC5 A8*, *FFAR2*, *FFAR3*, *NR1H4*, *S1PR2*, and *HIF1 A*) had similar expression pattern across the lifespan except for the low expression in the first trimester. The *FFAR2* gene was expressed by fetal enteroendocrine (EEC) and goblet cells as well as ${\text M}\upphi$ and ILC3 s, while the *FFAR3* gene was only expressed by fetal ${\text M}\upphi$. The *NR1H4* gene was only expressed by IEC. The *RORC* gene was predominantly expressed by fetal ILCs, and *HIF1 A* was ubiquitously expressed in all epithelial and immune cells.

## Discussion

Defining when the host-microbial interactions are first established is critical for understanding normal development and identifying disease preventive strategies. Recent research has highlighted the importance of the first 3 years of life as critical for immune system development [4]. With the recent identification of memory T cells in fetal tissues [5–8, 10, 11], the question arises whether intestinal T-cell antigens or other microbial components exposure and host-microbiome interaction begin in utero, prior to delivery.

To explore in utero immune function, we had previously sought to identify potential antigens recognized by fetal intestinal T cells, but the detection of the microbiome in fetal intestines or meconium was unsuccessful [30]. This is consistent with findings from other groups [15–19] who had also failed to identify microbiota in gestational tissues, which all suggest that there is an absence of live microbiota in the fetal tissue. However, this does not rule out that microbial components cannot be found in utero. Building on murine studies that found maternally derived bacterial byproducts in murine fetal intestines drive immune and epithelial development [29], we previously applied the metabolomic analysis pipeline from Metabolon and provided a comprehensive report of the in utero human intestinal metabolome with an abundance of bacterially derived metabolites and aryl hydrocarbon receptor (AHR) ligands implicated in mucosal immune regulation [30]. Given all these findings, in the current study, we hypothesized that microbiome-associated metabolites detected in fetal intestines in utero are primarily derived from the maternal microbiota and then travel to the human fetal intestine. To test this hypothesis, we examined a human cohort of pregnancy-matched fetal tissue including the fetal intestine, the fetal meconium, the fetal placental villi, and the maternal decidua.

Consistent with our previous work, we found that the microbial metabolites (microbially derived or bacteria associated metabolites) were present in the fetal intestine and across all the tissues examined. Furthermore, we found that the metabolomic profile of fetal intestinal tissue was distinct from that of the fetal meconium, the fetal placental villi, and the maternal decidua and contained bacterial metabolites. Focusing on the fetal GI tract, the top 20 most abundant metabolites contained a bacterially produced aromatic lactic acid, dihydrocaffeic acid (DHCA), which has been associated with decreasing intestinal inflammation [40–42]. By ingenuity pathways analysis (IPA), we further identified numerous pathways that were enriched for in the GI track compared to all other tissues that included transport of bile acids and inorganic cations, phase II conjugation of compounds, and tryptophan catabolism. Tryptophan serves as the only precursor for serotonin synthesis that occurs predominantly in the intestine by enteroendocrine cells (EEC) [43] where it plays many important functions including motility, secretion, and visceral sensitivity. When compared to meconium, additional signaling/endocrine pathways were upregulated in the fetal intestine including neurotransmitter release cycle, serotonin receptor signaling, arachidonic acid metabolism, prostaglandins and thromboxanes synthesis, and insulin secretion. We have previously demonstrated that the EEC in the fetal small intestine produce insulin and all the necessary machinery for glucose sensing and insulin secretion [31]. Transport of bile acids and inorganic cations is a known function of enterocytes, and these data suggest that these pathways are active even in utero. This was further supported by the transcriptomics data that demonstrated that many of the bile acid transporters and receptors were expressed in utero. Phase II conjugation is a detoxifying step in drug and toxin metabolism that occur in the GI tract and liver, and this data similarly suggests that this process is active in the enterocytes in utero. Interestingly, when compared to the meconium, many metabolic pathways were upregulated in the fetal GI tract, suggesting that fetal enterocytes are playing an active role in the absorption of amniotic fluid in utero. For example, glutamate metabolism is critical to the GI tract, where glutamate has been shown to be the largest contributor to intestinal energy generation and is a precursor for glutathione, arginine, and proline in the small intestine [44].

Although live fetal microbiome does not exist in utero, maternal microbiome is important to fetal intestinal and immune development [12, 14]. Data suggests that neonatal mice born to mothers transiently colonized with bacteria have increased xenobiotic metabolic signatures in their intestines compared to germ-free (GF) pups and contained traces of maternal microbiome metabolites in their intestines [29]. Additionally, work from the Elaine Hsiao’s group demonstrated that the maternal intestinal microbiome promotes placental development where depletion of intestinal microbiome restricted placental growth, and this was partially driven by SCFA [45]. The effects on placenta were also studied in [14]. The correlation analysis performed in this study suggests that the microbial metabolites detected in fetal samples were more likely to be vertically transmitted from maternal microbiota, because the metabolites present in the fetal GI and decidua samples had substantially higher positive correlation than the correlation between the fetal GI and meconium samples, either based on subject-matched samples or all samples analyzed together. We also investigated the variability of the abundance of the various metabolites in different tissues. Our data show that the secondary bile acid, lithocholic acid, the aromatic lactic acid, 5-MIAA, and another nine microbial metabolites were significantly enriched for in the decidua samples. Interestingly, many microbial metabolites were found in similarly abundance across all tissue samples.

Importantly, three different SCFA, butyric, isovaleric, and propionic acids, were found in fetal tissue with propionic acid being enriched for in the fetal GI tract. Butyric acid is an important energy source for enterocytes [35, 36], perhaps explaining why its levels were reduced in the GI compared to other tissue. It also plays important roles in enterocyte proliferation, differentiation, and maturation [35, 36]. SCFA have been shown to have anti-inflammatory roles within the intestine through binding to GPR41/43 (G protein-coupled receptors, *FFAR3/2*) [46]. Both *FFAR2* and *3* were expressed in the fetal intestine where *FFAR2* was found on EEC, Goblet cells, ${\text M}\upphi$, and NK cells, while *FFAR3* was found predominantly in ${\text M}\upphi$. Secondary bile acids have also been shown to play beneficial roles in intestinal homeostasis, including regulating inflammation [47, 48]. A number of genes associated with bile acid or SCFA transport and signaling were expressed in utero, especially in the subtypes of epithelial and immune cells, and steadily increased from first trimester through adulthood. It is intriguing to speculate that the maternal microbiota supports the anti-inflammatory intestinal milieu of the neonatal intestine required to induce tolerance in the setting of rapid microbiome acquisition in early life.

The main limitations of this study were the relatively small sample size and the lack of other maternal tissues including blood or stool. Though we had samples from multiple fetal tissues and maternal decidua, we were unable to secure all matched samples. Thus, the conclusions drawn about the origin of the microbial byproducts from unmatched samples may be less robust. In the future, it would be informative to collect maternal fecal and blood samples matched to placental samples for direct correlation between the maternal intestinal microbiome and bacterial metabolites in the fetal tissue. Additionally, we did not have sufficient material to validate all metabolites discussed in the manuscript, and future validation studies should be conducted particularly for those that were identified as Level 3 (see details in Supplementary Table S9).

## Methods

### Sample collection

Placental and fetal samples were obtained from the University of Pittsburgh Biospecimen Core from electively terminated products of conception (14–23 weeks of gestation) with Institutional Review Board (IRB) approval and signed informed consent (IRB no. 18010491, University of Pittsburgh). Products of conception were collected from dilation and evacuation procedures with nonpharmacological, mechanical dilation via Dilapan-S. No fetal subject had reported genetic abnormalities. In respect of patient confidentiality and safety, limited clinical information was collected for fetal samples. All demographic information that could be legally and respectfully obtained is shown in **Supplementary** **Table S1**. After receiving fetal samples, the meconium was removed, and a small piece was cut with a sterile blade and immediately snap-frozen and stored at − 80 °C until processing. Samples were shipped on dry ice to Khatib laboratory for metabolomics analysis.

### Untargeted metabolomics analysis

#### Extraction method

All samples were weighed, and LC/MS grade methanol was added (1:4 w/v). The tissues were homogenized for 1 min using IKA T 18 digital ULTRA-TURRAX®. The samples were vortexed for 10 min in room temperature and centrifuged for 10 min with 15,294 g at 4 °C. Then the supernatant was filtered through 0.22-µm PTFE syringe filters (Membrane Solutions, USA) into HPLC vials and injected to LCMS.

#### LCMS analysis

The extracted solutions (5 μL) were injected into a UPLC connected to a photodiode array detector (Dionex UltiMate 3000), with a reverse-phase column (ZORBAX Eclipse Plus C18, 100 × 3.0 mm, 1.8 μm). The mobile phases consisted of phase A DDW with 0.1% formic acid and phase B acetonitrile containing 0.1% formic acid. The gradient was started with 98% A and increased to 30% B in 4 min and then increased to 40% B in 1 min and kept isocratic at 40% B for another 3 min. The gradient increased to 50% in 6 min, increased to 55% in 4 min, and finally increased to 95% in 5 min and kept isocratic for 7 min. Phase A was returned to 98% A in 3 min, and the column was allowed to equilibrate at 98% A for 3 min before the next injection. The flow rate was 0.4 mL/min. MS analysis was performed with HESI-II source connected to a Q Exactive Plus Hybrid Quadrupole-Orbitrap Mass Spectrometer from Thermo Fisher Scientific. ESI capillary voltage was set to 3500 V, capillary temperature to 300 °C, gas temperature to 350 °C, and gas flow to 10 mL/min. The mass spectra (m/z 100–1500) were acquired in negative ion mode (ESI −).

Blank and quality control (QC) samples were analyzed throughout the entire experimental procedure. Blank vials consisted of methanol. The QC samples were prepared by mixing 50 μL of each sample. Blank and QC samples were injected first in the sequence, after each set of 10 samples, and at the end of the sequence, to monitor the stability and performance of the system and evaluate the quality of the acquired data. Blank peaks were removed from the dataset of compounds, and normalization was performed using a linear regression model for QC correction.

### Metabolomic data acquisition using Compound Discoverer software

The LC–MS/MS data were analyzed using Compound Discoverer software (version 3.3.0.305; Thermo Scientific, Waltham, MA, USA). The identification was based on using the mzCloud database (https://www.mzcloud.org, based on MS2 spectra) and the ChemSpider database (https://www.chemspider.com/, based on MS1 scan). Metabolites identified by ChemSpider by MS1 without MS2 are labeled as Level 4 identification. Metabolites detected by ChemSpider with MS2 are labeled as Level 3 identification. Metabolites detected by mzCloud best match are labeled as Level 2. Metabolites validated by standards are labeled as Level 1 (hippuric acid, benzoate (benzoic acid), taurocholic acid (sodium taurocholate), and deoxycholic acid). Most of the pharmaceuticals identified are annotated as Level 4 without MS, which will need more validation using standards in the future. Results were normalized by incorporating QC samples throughout the extraction stages to check for repeatability and the extraction and normalization of the deviations. The QC samples were injected during all the stages of the run to test the stability and sensitivity of the devices and to normalize these deviations. Supplementary Table S8 includes all the metabolites detected in the samples with information about MW, RT values, and if the metabolites were detected based on MS2 or just by MS1 scan, mzCloud best match, and alternative options for the metabolite’s names especially those that identified just by MS1. The metabolites were divided into four sheets depending on the level of annotation: Level 1, metabolites identified using standards; Level 2, metabolites annotated using MS2 with mzCloud best match higher than 60%; Level 3, metabolites annotated using MS1; and Level 4, unknowns. Among the 2521 metabolites analyzed in this paper, 9 were identified as Level 1, 402 as Level 2, and 2110 as Level 3. Details of the metabolites discussed in the manuscript have been added to the new Supplementary Table S9.

### Quantitative metabolites analysis for validation

#### Data preprocessing

The standards and samples were injected using the same LC–MS method reported at the untargeted metabolomics part. Peak determination and peak area integration were performed with QuanBrowser (Thermo Xcalibur, version 4.1.31.9). Autointegration was manually inspected and corrected if necessary. Calibration curves were used for the quantification of each compound. Linear curves were obtained for all compounds with R2 > 0.99: hippuric acid 0.1–5000 ppb, benzoic acid 500–50,000 ppb, taurocholate 100–50,000 ppb, and deoxycholic acid 0.1–100 ppb.

### Method validation

Method validation was performed to determine the limit of detection (LOD), limit of quantitation (LOQ), linearity repeatability, and recovery for each compound.

For intraday precision, a mixture of all the metabolites standards was prepared and injected as QC at the beginning of the sequence, then after each 10 samples, and at the end of the sequence. The RSDs into QC samples were calculated for each analyte to be less than 6.6%.

For recovery analysis, three samples were spiked, extracted, and injected to LCMS. The concentration of each analyte was calculated into the spiked and non-spiked samples, and the recovery was evaluated to be on average 82% for benzoic and hippuric acids, 92% for taurocholate, and 98% for deoxycholic acid.

LOD and LOQ were determined by signal-to-noise ratios higher than 3 and 10, respectively. LOD and LOQ for hippuric acid, deoxycholic acid, and sodium taurocholate were 0.1 ppb. LOD and LOQ for benzoic acid were 500 ppb.

### Metabolomic data analysis

#### Data preprocessing

The data preprocessing steps for the main data set (a) and another two validation data sets (b–c) are introduced in the following parts respectively.

(a) The original metabolome data matrix contained 65 samples and 18,424 compounds. The data were preprocessed according to Li, Yujia et al. [30]. The blank samples, quality control samples, and one sample without location information were filtered out, resulting in 49 samples (8 decidua, 8 small intestine, 3 large intestine, 11 small intestine meconium, 8 large intestine meconium, and 11 PV). Compounds without names (based on the protocol for annotation described above) were discarded, and for the named compounds with isomers, the one with the largest inter-quantile range over the 49 samples at log scale was kept. Next, the resulting metabolome data matrix containing 49 samples and 2521 metabolites were log-transformed (base 2) and normalized across samples by quantile normalization using the “preprocessCore” R package [49].

(b) Due to sample limitation where no additional sample was available, metabolites were validated by standards without the exact concentrations of the samples calculated. This raw validation metabolome data matrix contained 65 samples (same as original data matrix) and 5 compounds. After filtering out the blank samples, quality control samples, and one sample that failed QC, the remaining 49 samples and 5 metabolites were log-transformed (base 2).

(c) Where additional samples were available, metabolites were validated by standards, and exact concentrations were calculated. This raw validation data set included the quantitative abundance of 4 metabolites and 55 samples. Samples with abnormal quantity or without tissue location information were filtered out, resulting in 48 samples remaining for hippurate, 52 samples for benzoate, 48 samples for taurocholic acid, and 50 samples for deoxycholic acid. Missing values were imputed by assigning half of the minimum observed value of each metabolite and addition of a small random noise.

### Determination of metabolite clustering

*t*-SNE (R package “Rtsne” [50–52]) plots were made to visualize the separability of samples from different tissue locations. For the specific values of the t-SNE parameters, we set the perplexity to be 10, the number of iterations to be 5000, and all the other parameters as default values.

### Statistical and bioinformatic analyses

The statistical analysis includes five parts with details described in the following sections respectively: differential analysis (a), pathway enrichment analysis (b), correlation analysis (c), ANOVA analysis (d), and gestational age association analysis (e).

(a) For differential analysis, the “limma” R package [53] was used to detect the differentially expressed metabolites and pathways in three pairwise comparisons: decidua versus GI (small intestine and large intestine), GI versus meconium, and GI versus PV. We applied “limma” R package to calculate *p*-values and log-fold changes for individual metabolites, followed by Benjamini–Hochberg procedure to correct for multiple testing, to control false discovery rate, and to report *q*-value. The results were visualized in volcano plots using the “EnhancedVolcano” R package [54] where the significantly differentially expressed genes are highlighted in red with the absolute value of log fold change larger than 2.5 and *q*-value smaller than 0.05.

(b) For pathway enrichment analysis, we figured out the KEGG ID of 843 metabolites. Based on the KEGG IDs and identified differentially expressed metabolites, pathway enrichment analysis for each of the three pairwise comparisons was conducted using QIAGEN Ingenuity Pathway Analysis (IPA) software for metabolome data (https://www.qiagenbioinformatics.com/products/ingenuitypathway-analysis) [55]. This tool generated *q*-values and enrichment effect sizes (log odds ratio).

(c) The correlation matrix provided the pairwise correlations of the six tissue groups (decidua, small intestine (SI), large intestine (LI), small intestine meconium (SI Mec), large intestine meconium (LI Mec), and placental villi (PV)) from subject no. 16 based on the abundance of 41 bacterially derived metabolites and was visualized by the “ggcorrplot” R package [56]. In the boxplots, each point is the abundance correlation of microbial metabolites/xenobiotic/fetal-derived metabolites between one decidua sample and one GI sample (or one GI sample and one meconium sample). The significance levels were calculated by two sample *t*-test.

(d) For each microbial metabolite, bile acid and aromatic acid, we performed one-factor ANOVA and post hoc analysis to compare the difference of its abundance among the four tissue groups (decidua, GI, meconium, and PV). For each bile acid, we further performed ANOVA analysis adjusted by the gestational age, i.e., involving gestational age as confounder. The results were visualized in heatmap by “pheatmap” R package [57] and boxplots generated by the “ggplot2” package [58].

(e) To investigate the association between the abundance of individual metabolite with gestational age and tissue group for bile acids and SCFAs, we applied the linear regression model using “limma” R package, reporting the coefficient slopes and *q*-values.

### Single-cell RNA-seq data analysis

The single-cell data was from one of our previous published single-cell atlas of human small intestine throughout the human lifespan [39]. The fetal epithelial cells and immune cells were clustered using Scanpy (v1.9.2) package [59] as described in Gu et al. [39]. Briefly, gene expression in each cell was normalized and log transformed. Afterwards, highly variable genes were identified using the scanpy.pp.highly_variable_genes function with default parameters. In addition, the effects of the percentage of mitochondrial genes, the percentage of ribosomal protein genes, and the unique molecular identifier (UMI) counts were regressed out using scanpy.pp.regress_out function before scaling the data. Batch correction of samples was performed with bbknn (v1.5.1) [60]. Dimensionality reduction and Leiden clustering were carried out on the remaining highly variable genes, and the cells were visualized using Uniform Manifold Approximation and Projection (UMAP) plots. Cell types were manually annotated based on the known marker genes found in the literature. The statistical analysis of the mean expression values of the selected genes in each detected cell in each sample was performed using one-way ANOVA with Tukey’s multiple comparison test to compare gene expression among developmental stages. The analysis was conducted using GraphPad Prism 9, and differences were considered statistically significant at a *p*-value < 0.05.

## Supplementary Information

Supplementary Material 1: Supplementary Figure S1. t-distributed stochastic neighbor embedding (t-SNE) plot using all metabolites. The samples are color-coded by different tissue sites, with different letter shapes according to their source subjects. Those subjects that only have one sample are labelled as “Others”. Supplementary Figure S2. Distribution of gestational agein samples (A) The density curves of gestational age for samples within each of the four groups. (B) Boxplots of gestational age for each group where each black dot represents a sample. The median sample age shown on the top is 22.5 weeks for decidua, 18.5 weeks for GI, 21 weeks for meconium and 22 weeks for PV group respectively. The pairwise difference among the four groups is not significant (i.e., all adjusted *P*-value > 0.05) by one-factor ANOVA and post-hoc analysis. Supplementary Figure S3. Correlation between tissue groups of microbial metabolites, xenobiotics, and fetal metabolites. Correlation between tissue groups of microbial metabolites, xenobiotics, and fetal metabolites. Boxplots visualizing the correlations between GI and decidua samples and between GI and meconium samples from the subjects at gestational age of 23 weeks, based on 41 microbial metabolites, 47 xenobiotics, and 8 fetal-derived metabolites respectively from Supplementary Table S5. Red asterisk points represent the pairwise correlations between tissue samples from subject No. 16. Supplementary Figure S4. Metabolites validation. Boxplots in the first row show the abundance difference between tissue groups for the eight metabolites in the main data set, while the second and third rows present the difference in validation data sets for the corresponding metabolites. Supplementary Figure S5. Markers for cell type annotation. Dot plot of the marker genes for the annotation of fetal epithelial cells (A) and of fetal immune cells (B). Color represents normalized mean expression of marker genes in each cell type, and size indicates the proportion of cells expressing marker genes. Supplementary tables: Table S1. Sample demographics. Demographic information for all samples used. N/A, not available; SI, small intestine; LI, large intestine; SI Mec, small intestine meconium; LI Mec, large intestine meconium; F, female; M, male. Supplementary Table S2. List of the detectable metabolites and their abbreviations. Supplementary Table S3. Difference of metabolite abundance in pairwise comparisons between GI and the other tissue groups. Supplementary Table S4. Enriched pathways in pairwise comparisons between GI and the other tissue groups. Supplementary Table S5. List of microbial metabolites, xenobiotics, and fetal-derived metabolites used in the analysis. * indicates the microbial metabolites reported by (30) (Supplementary Tables S8, S9), and † indicates the identified microbial metabolites based on (32, 33). Supplementary Table S6. Correlation coefficients and significance levels (*p* value and q value) of gestational age for each bile acid by ANOVA analysis with adjustment of gestational age. Supplementary Table S7. Correlation coefficients between the abundance of individual metabolites and the gestational age within each tissue group. Except for the bile acids and SCFA in Table 2[1], showing all the other nine bile acids and two SCFAs. The columns are the correlation coefficient within each tissue group separately. The q value for the correlation coefficient shows in parathesis *P < 0.05, **P < 0.01, ***P < 0.001. Supplementary Table S8. Comprehensive information of all metabolites detected in the samples. The table contain all the metabolites detected in the samples with information about MW, RT values, and if the metabolites detected based on MS2 or just by MS1 scan, MzCloud best much, as well as alternative options for the metabolite's names especially those that identified just by MS1. Supplementary Table S9. Information on metabolites discussed in the manuscript. Identification information of discussed metabolites including microbial metabolites (secondary bile acid, SCFA, aromatic lactic acids, and others), xenobiotics, fetal-derived metabolites, primary bile acids, aromatic amino acids, top 20 abundant metabolites in GI tract in Fig. 1B, and DE metabolites labelled on volcano plots of Fig. 1 C-E CE: This citation is not found in floats.Please check if it is citation or not and proceed further

## Data Availability

No datasets were generated or analysed during the current study.
