# Supplementary material for: In utero human intestine contains maternally derived bacterial metabolites
Source: Microbiome. 2025 May 6;13:116. doi: 10.1186/s40168-025-02110-0 (PMC12054239; doi:10.1186/s40168-025-02110-0)

Supplementary Material

In utero human intestine contains maternally derived bacterial metabolites

Wenjia Wang[1], Weihong Gu[2], Ron Schweitzer[3,4,5], Omry Koren[3], Soliman Khatib[4,5], George Tseng[1], Liza Konnikova[2,6-10]

[1]Department of Biostatistics, University of Pittsburgh, Pittsburgh, PA, USA

[2]Department of Pediatrics, Yale School of Medicine, New Haven, CT, USA

[3]Azrieli Faculty of Medicine, Bar Ilan University, Safed, Israel

[4]Department of Biotechnology, Tel-Hai College, Upper Galilee, Israel

[5]Department of Natural Compounds and Analytical Chemistry, Migal Galilee Research Institute, Kiryat Shmona, Israel

Departments of [6]Immunobiology and [7]Obstetrics, Gynecology, and Reproductive Sciences; [8]Program in Translational Biomedicine; [9]Human Translational Immunology Program; and [10]Center for Systems and Engineering Immunology, Yale School of Medicine, New Haven, CT, USA.

Supplementary Table Legends

***Supplementary Table S1.* Sample demographics.** Demographic information for all samples used. N/A, not available; SI, small intestine; LI, large intestine; SI Mec, small intestine meconium; LI Mec, large intestine meconium; F, female; M, male.

Supplementary Table S2. List of the detectable metabolites and their abbreviations.

Supplementary Table S3. Difference of metabolite abundance in pairwise comparisons between GI and the other tissue groups.

Supplementary Table S4. Enriched pathways in pairwise comparisons between GI and the other tissue groups

Supplementary Table S5. List of microbial metabolites, xenobiotics, and fetal-derived metabolites used in the analysis. * indicates the microbial metabolites reported by (30) (**Supplementary Tables S8, S9**), and † indicates the identified microbial metabolites based on (32, 33).

Supplementary Table S6. Correlation coefficients and their significance levels (p-value and q-value) of gestational age for each bile acid by ANOVA analysis with adjustment of gestational age.

Supplementary Table S7. Correlation coefficients between the abundance of individual metabolites and the gestational age within each tissue group. Except for the bile acids and SCFA in Table 2, showing all the other nine bile acids and two SCFAs. The columns are the correlation coefficient within each tissue group separately. The q value for the correlation coefficient shows in parathesis *P < 0.05, **P<0.01, ***P<0.001.

Supplementary Table S8. Comprehensive information of all metabolites detected in the samples. The table contain all the metabolites detected in the samples with information about MW, RT values, and if the metabolites detected based on MS2 or just by MS1 scan, MzCloud best much, as well as alternative options for the metabolite's names especially those that identified just by MS1.

Supplementary Table S9. Information o*n* metabolites d*iscussed in the manuscript*. Identification information of discussed metabolites including microbial metabolites (secondary bile acid, SCFA, aromatic lactic acids, and others), xenobiotics, fetal-derived metabolites, primary bile acids, aromatic amino acids, top 20 abundant metabolites in GI tract in Figure 1B, and DE metabolites labelled on volcano plots of Figure 1C-E.

Supplementary Figure Legends

Supplementary Figure S1. t-distributed stochastic neighbor embedding (t-SNE) plot using all metabolites. The samples are color-coded by different tissue sites, with different letter shapes according to their source subjects. Those subjects that only have one sample are labelled as “Others”.

Supplementary Figure S2. Distribution of gestational age in samples. (A) The density curves of gestational age for samples within each of the four groups. (B) Boxplots of gestational age for each group where each black dot represents a sample. The median sample age shown on the top is 22.5 weeks for decidua, 18.5 weeks for GI, 21 weeks for meconium and 22 weeks for PV group respectively. The pairwise difference among the four groups is not significant (i.e., all adjusted P-value > 0.05) by one-factor ANOVA and post-hoc analysis.

Supplementary Figure S3. Correlation between tissue groups of microbial metabolites, xenobiotics, and fetal metabolites. Boxplots visualizing the correlations between GI and decidua samples and between GI and meconium samples from the subjects at gestational age of 23 weeks, based on 41 microbial metabolites, 47 xenobiotics, and 8 fetal-derived metabolites respectively from Supplementary Table S5. Red asterisk points represent the pairwise correlations between tissue samples from subject No. 16.

Supplementary Figure S4. Metabolites validation. Boxplots in the first row show the abundance difference between tissue groups for the eight metabolites in the main data set, while the second and third rows present the difference in validation data sets for the corresponding metabolites.

Supplementary Figure S5. Markers for cell type annotation. Dot plot of the marker genes for the annotation of fetal epithelial cells (A) and of fetal immune cells (B). Color represents normalized mean expression of marker genes in each cell type, and size indicates the proportion of cells expressing marker genes.

Supplementary Table S1.

Subject No.	Tissue type	Gestational age (weeks)	Sex
1	Dec, LI Mec, SI Mec, PV	20	F
2	SI	16-17	N/A
3	SI	16	N/A
4	SI	17-18	N/A
5	SI	23	N/A
6	SI	18	N/A
7	SI	19-20	N/A
8	PV	21	N/A
9	PV	21	F
10	Dec, PV	22	F
11	SI, LI Mec, SI Mec	19	F
12	Dec, LI Mec, SI Mec, PV	23	F
13	Dec, LI Mec, SI Mec, PV	22	M
14	SI Mec	23	N/A
15	Dec, SI Mec, PV	23	N/A
16	Dec, SI, LI, LI Mec, SI Mec, PV	23	F
17	SI Mec	14	M
18	SI Mec, LI Mec	21	M
19	LI	15	N/A
20	SI Mec, PV	18	M
21	Dec, LI Mec, PV	23	F
22	LI, LI Mec	18-19	M
23	Dec, SI Mec, PV	21	N/A

Supplementary Table S5.

Bacterial Metabolites	Xenobiotic Metabolites	Fetal-Derived Metabolites
* Pyridoxine	Penicillin-G	N-Acetylaspartylglutamicacid
* Pantothenic acid	11-nor-9-Carboxy-Delta-9-THC	5α-Pregnan-3,20-dione
N-Acetyl-alpha-D-glucosamine1-phosphate	(+)-Simvastatin	Pregnenolonesulfate
* Riboflavin	(-)-Abietic acid	Homoarginine
* Pipecolic acid	(-)-Citronellol	N-Acetylcadaverine
alpha-L-Arabinose	(-)-Febrifugine	17-Hydroxypregnenolone sulfate
Pantetheine	(-)-Rhododendrin	1beta-Hydroxycholic acid
2,2'-Methylenebis(6-tert-butyl-p-cresol)	(+)-10-Deoxymethynolide	Androsterone
* Kynurenine	(+)-N-Methylconiine	
* Benzoate	(+/-)-Methoprene	
MTA	(–)-trans-Methyl dihydrojasmonate	
Methylhippuricacid	(1S,2R,3R,7R)-7-Isopropenyl-1-methyl-1,2,3,4,5,6,7,8-octahydro-2,3-naphthalenediol	
Coprocholic acid	(1S,2S,8aR)-1-[(3Z)-5-hydroxy-3-methylpent-3-en-1-yl]-2,5,5,8a-tetramethyl-decahydronaphthalen-2-ol	
4-Hydroxyphenacyl alcohol	[Similar to: Nodularin; ΔMass: -623.3793 Da]	
albaflavenol	18-acetoxy-1alpha-hydroxyvitamin D3	
1H-Indole	18-acetoxy-1alpha,25-dihydroxyvitamin D3	
2-(4,6-diphenyl-1,3,5-triazin-2-yl)-5-(hexyloxy)phenol	2-(4-Hydroxyphenyl)ethanol(Tyrosol)	
4-vinylphenol sulfate	2-(4-Nonylphenoxy)ethanol	
DMDBS	2-{2-[2-(Decyloxy)ethoxy]ethoxy}ethanol	
Phosphopantothenic acid	Asparenomycin A	
* Phenol	Dehydrocholic acid	
* Hippurate	Adaprolol	
* Biotin	Alizapride	
Butoxyethyl phthalate	alpha-Tocotrienol	
Butyl-o-cresol	Ascorbyl stearate	
Bis(4-ethylbenzylidene)sorbitol	Cannabidiol	
* Thiamine	Carvedilol	
* p-Cresol	Cymoxanil	
2-Hydroxyhippuric acid	Cyprodenate	
† 5-MIAA	Doxycycline	
* Indoxyl sulfate	Elacytarabine	
* Deoxycholic acid	Eglumetad	
Glycodeoxycholic acid	Gabapentin	
Sulfolithocholic acid	Guaifenesin	
* Taurodeoxycholic acid	ibufenac	
* Lithocholic acid	Imiprothrin	
* Butaric acid	Ipratropium	
Isovaleric acid	Ketamine	
Propionic acid	Ketorolac	
† Dihydrocaffeic acid or DHCA	Mepivacaine	
† Ethylparaben	Mescaline	
	Morphine	
	Nifedipine	
	pentobarbital	
	Perindopril	
	Picaridin	
	Rimexolone	

Supplementary Table S6.

Bile Acid	GA Effect Size	p-value	q-value
Glycodeoxycholic acid	0.119857	0.292251	1
Sulfolithocholic acid	0.21543	0.156541	1
Deoxycholic acid	-0.05175	0.642545	1
Taurodeoxycholic acid	0.013903	0.882138	1
Lithocholic acid	-0.06723	0.150412	1
Glycocholic acid	0.136196	0.40203	1
Chenodeoxyglycocholic acid	0.071728	0.586829	1
Glycochenodeoxycholic acid	0.229687	0.189903	1
7-Sulfocholic acid	0.039478	0.768244	1
Muricholic acid	0.044299	0.75679	1
Taurocholic acid	0.324754	0.062937	0.881119
Taurochenodeoxycholic acid	0.344618	0.084407	1
1beta-Hydroxycholic acid	0.057559	0.569992	1
3α,7α,12β-trihydroxy-5β-cholanic acid	0.023966	0.763369	1

Supplementary Table S7.

	Decidua	GI	Mecomium	PV
Glycochenodeoxycholic acid	0.01<br>(0.99)	-0.17<br>(0.36)	0.39<br>(0.43)	-0.19<br>(0.96)
Chenodeoxyglycocholic acid	0.45<br>(0.94)	0.10<br>(0.47)	-0.02<br>(0.92)	-0.02<br>(0.96)
Glycocholic acid	0.51<br>(0.94)	-0.13<br>(0.46)	0.13<br>(0.76)	0.17<br>(0.96)
7-Sulfocholic acid	0.35<br>(0.94)	0.19<br>(0.11)	0.05<br>(0.92)	0.19<br>(0.96)
Taurocholic acid	-0.41<br>(0.94)	-0.13<br>(0.47)	0.33<br>(0.46)	-0.03<br>(0.96)
Taurodeoxycholic acid	-0.18<br>(0.94)	0.04<br>(0.71)	0.06<br>(0.82)	0.02<br>(0.96)
Sulfolithocholic acid	0.18<br>(0.94)	0.25<br>(0.06)	0.32<br>(0.43)	0.06<br>(0.96)
Lithocholic acid	-0.05<br>(0.94)	0.03<br>(0.61)	-0.06<br>(0.73)	-0.11<br>(0.96)
Taurochenodeoxycholic acid	-0.17<br>(0.94)	-0.09<br>(0.69)	0.50<br>(0.43)	-0.09<br>(0.96)
Isovaleric acid	0.08<br>(0.94)	0.01<br>(0.90)	0.02<br>(0.92)	0.23<br>(0.96)
Butyric acid	-0.06<br>(0.94)	0.00<br>(0.99)	-0.10<br>(0.73)	0.19<br>(0.96)

Supplementary Figure S1.

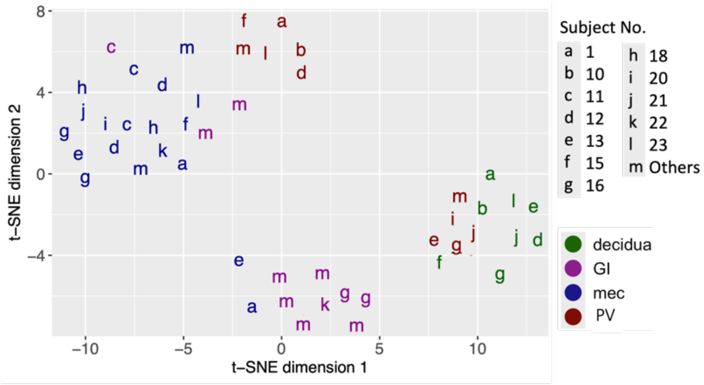

Supplementary Figure S2.

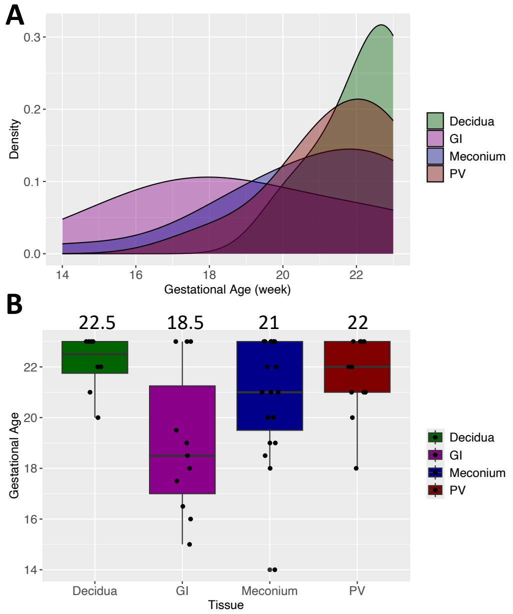

Supplementary Figure S3.

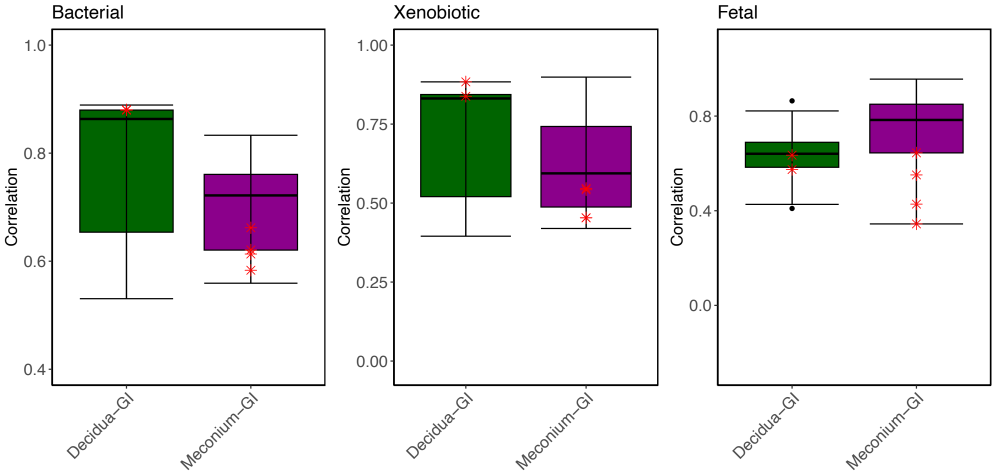

Supplementary Figure S4.

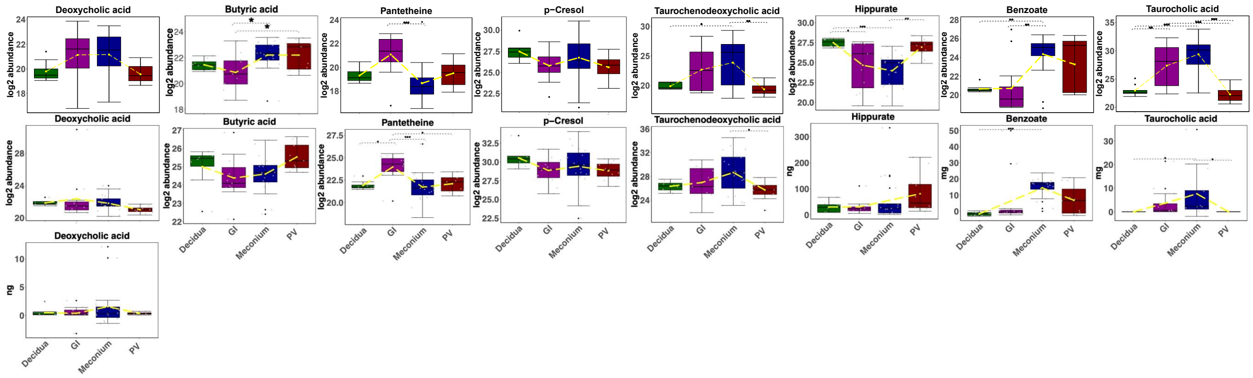

Supplementary Figure S5.

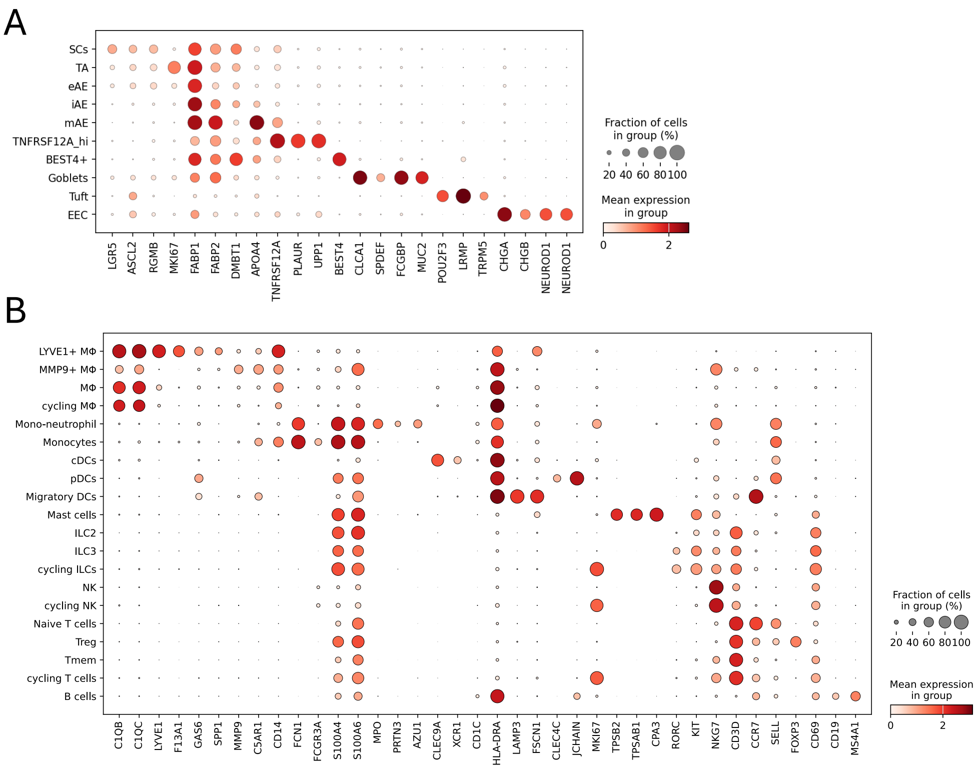

Supplement: Supplementary file 2 — Supplementary Material 1: Supplementary Figure S1. t-distributed stochastic neighbor embedding (t-SNE) plot using all metabolites. The samples are color-coded by different tissue sites, with different letter shapes according to their source subjects. Those subjects that only have one sample are labelled as “Others”. Supplementary Figure S2. Distribution of gestational agein samples (A) The density curves of gestational age for samples within each of the four groups. (B) Boxplots of gestational age for each group where each black dot represents a sample. The median sample age shown on the top is 22.5 weeks for decidua, 18.5 weeks for GI, 21 weeks for meconium and 22 weeks for PV group respectively. The pairwise difference among the four groups is not significant (i.e., all adjusted P-value > 0.05) by one-factor ANOVA and post-hoc analysis. Supplementary Figure S3. Correlation between tissue groups of microbial metabolites, xenobiotics, and fetal metabolites. Correlation between tissue groups of microbial metabolites, xenobiotics, and fetal metabolites. Boxplots visualizing the correlations between GI and decidua samples and between GI and meconium samples from the subjects at gestational age of 23 weeks, based on 41 microbial metabolites, 47 xenobiotics, and 8 fetal-derived metabolites respectively from Supplementary Table S5. Red asterisk points represent the pairwise correlations between tissue samples from subject No. 16. Supplementary Figure S4. Metabolites validation. Boxplots in the first row show the abundance difference between tissue groups for the eight metabolites in the main data set, while the second and third rows present the difference in validation data sets for the corresponding metabolites. Supplementary Figure S5. Markers for cell type annotation. Dot plot of the marker genes for the annotation of fetal epithelial cells (A) and of fetal immune cells (B). Color represents normalized mean expression of marker genes in each cell type, and size indicat [file 40168_2025_2110_MOESM1_ESM.pdf]
